# Constitutive Equations for Describing the Hot Compressed Behavior of TC4–DT Titanium Alloy

**DOI:** 10.3390/ma13153424

**Published:** 2020-08-03

**Authors:** Haoran Wang, Chunge Wang, Muyu Li, Rui Ma, Jun Zhao

**Affiliations:** 1 College of Mechanical Engineering, Yanshan University, Qinhuangdao 066004, China; hrwang@stumail.ysu.edu.cn (H.W.); limuyu@stumail.ysu.edu.cn (M.L.); mar@ysu.edu.cn (R.M.); 2 Key Laboratory of Advanced Forging & Stamping Technology and Science (Yanshan University), Ministry of Education of China, Qinhuangdao 066004, China; 3 College of Mechanical and Energy Engineering, Ningbo Institute of Technology, Zhejiang University, Ningbo 315100, China; wangchunge@nit.zju.edu.cn

**Keywords:** constitutive equation, modified Arrhenius-type equation, strain rate, the Hensel–Spittel (HS) equation

## Abstract

Isothermal hot compression tests of TC4–DT titanium alloy were performed under temperatures of 1203–1293 K and strain rates of 0.001–10 s^−1^. The purpose of this study is to develop a new high-precision modified constitutive model that can describe the deformation behavior of TC4–DT titanium alloy. Both the modified strain-compensated Arrhenius-type equation and the modified Hensel–Spittel equation were established by revising the strain rate. The parameters in the above two modified constitutive equation were solved by combining regression analysis with iterative methods, which was used instead on the traditional linear regression methods. In addition, both the original strain-compensated Arrhenius-type equation and Hensel–Spittel equation were established to compare with the new modified constitutive equations. A comparison of the predicted values based on the four constitutive equations was performed via relative error, average absolute relative error (AARE) and the correlation coefficient (R). These results show the modified Arrhenius-type equation and the modified Hensel–Spittel equation is more accurate and efficient with a similar prediction accuracy. The AARE-value of the two modified constitutive equation is relatively low under various strain rates and their fluctuation is small as the strain rate changes.

## 1. Introduction

The deformation behavior of alloys is complicated at elevated temperatures. The deformation behavior of alloys is affected by many factors, including deformation conditions, work hardening (WH), dynamic recovery (DRV) and dynamic recrystallization (DRX) and phase transition. Constitutive equations can represent the material behaviors during deformation, and these equations can provide a fundamental understanding of the metal forming process [1,2]. Because a constitutive equation can relate stress and strain to the related conditions of temperature and strain rate, it plays a crucial role in numeric analysis, modeling and optimizing hot forming process parameters [3].

There were three main constitutive model categories: phenomenological constitutive models, physical-based constitutive models and artificial neural network constitutive models, which are used to predict the constitutive behavior of metals and alloys [4]. Moreover, the phenomenological constitutive models have a lesser number of material constants and can be easily calibrated [4]. Among all the phenomenological constitutive models, the strain-compensated Arrhenius-type model is widely used. Based on the flow stress of modified 9Cr–1Mo steel and SnSbCu Alloy, it was found that the strain-compensated Arrhenius-type model has higher prediction accuracy than the Johnson-Cook model and modified Zerilli–Armstrong model [5,6]. To predict the hot flow stress of 28CrMnMoV steel, Li et al. [7] found the prediction accuracy of the strain-compensated Arrhenius-type model is higher than that of modified Johnson Cook, modified Zerilli–Armstrong equation. For describing the hot deformation behavior, Wang found the strain-compensated Arrhenius-type model had the highest accuracy when compared to Johnson–Cook model, modified Johnson–Cook model, Zerilli–Armstrong model, modified Zerilli–Armstrong model and KHL model [8]. A similar result was obtained by developing constitutive models of 21–4 N heat resistant steel [9]. Moreover, the strain-compensated Arrhenius-type equation is most widely applied to describe the high-temperature flow behavior of metals and alloys [10,11,12,13,14,15,16,17,18]. These results mean that the strain-compensated Arrhenius-type model has a relatively strong predictive ability.

About the Arrhenius-type equation, Sellars and Tegart proposed that the sine-hyperbolic law can be used over a very wide range of stress [19], which is shown in Equation (1). The common effect of the temperatures and strain rates on the hot deformation behavior can be shown through the Zener–Hollomon parameter (*Z*) in an exponent-type equation, which is shown in Equation (2) [17].

(1) $$\dot{\varepsilon }=AF(\sigma )\exp(-Q/(\mathrm{RT}))$$

(2) $$Z=\dot{\varepsilon }\exp(Q/(\mathrm{RT}))$$

where

(3) $$F(\sigma )=\sigma^{n_{1}}(\alpha \sigma <0.8)$$

(4) F(σ)=exp(βσ)(ασ>1.2)

(5) $$F(\sigma )={\left[\sinh(\alpha \sigma )\right]}^{n}(for\;all\;\sigma )$$

where $\dot{\varepsilon }$ is the real strain rate (s^−1^), *σ* is the flow stress (MPa), T is the deformation temperature (K), Q is the activation energy (J·mol^−1^), R is the molar gas constant (8.3145 J·mol^−1^·K^−1^), A, *n*_1_, *n*, *α* and *β* are material constants. Moreover, *α* = *β*/*n*_1_.

Because there is no strain in the Arrhenius-type equation, the polynomial is employed to represent the influence of strain on material constants to establish the strain-compensated Arrhenius-type equation [20,21]. Moreover, the material constant *α* is used as an adjustable parameter to improve the predictive power of the Arrhenius-type equation [22,23,24,25]. Moreover, the exponent of the strain rate in the Arrhenius-type equation was revised to give an accurate and precise estimate for the flow stress of 42CrMo steel [20]. Wang et al. [26] proposed to obtain a more reasonable value of material constants by combining the iterative methods and regression analysis.

Regarding the Hensel-Spittel equation, it was proposed by Hensel and Spittel to describe the hot deformation behavior of metals and alloys [27]. Wei et al. [28] used Hensel-Spittel equation to describe the hot deformation of Mg–9Li–3Al–2Sr–2Y under the whole compression process. Claudimir J et al. [29] established the constitutive model of HSLA350/440 and DP350/600 steels based on the Hensel–Spittel equation. Flora et al. [30] used the Hensel–Spittel equation to predict the flow stress of TiAl–Mo alloys at high temperatures.

Because of its excellent physical and mechanical properties, TC4–DT titanium alloy is extensively adopted in a wide range of temperature applications. The elevated temperature can improve the plastic deformation ability of TC4–DT titanium alloy. Therefore, the hot deformation behaviors of TC4–DT titanium alloy have been investigated by some researchers.

Based on the flow stress of TC4–DT titanium alloy, Liu et al. [31] found there was low prediction accuracy of the strain-compensated Arrhenius-type flow stress equation at the higher strain rate. Peng et al. [32] obtained a similar result. In addition, based on hot deformation behavior of Ti2AlNb-based alloys, He et al. [33] performed a comparative study and found the Arrhenius-type equation was not suitable for the deformation with relatively higher strain rates. However, Tao et al. [34] found that the application of the strain-compensated Arrhenius-type equation is limited by its relatively low predicted accuracy at lower strain rates. In addition, for describing the hot tensile deformation Behavior of TC4–DT alloy (also named Ti–6Al–4V Alloy), Lin et al. [35] found the prediction accuracy of the Hensel–Spittel equation is higher than that of the strain-compensated Arrhenius-type equation.

In summary, because the strain-compensated Arrhenius-type equation cannot consider the effect of strain rates on stress relatively accurately, the accuracy under specific strain rates is relatively lower for predicting the flow stress of some metals and alloys, especially TC4–DT alloy. Although Peng et al. [32] improved the prediction accuracy of the strain-compensated Arrhenius-type equation by modifying the temperature and the exponent of strain rate under partial deformation conditions, they did not attempt to propose an effective method to establish a simpler constitutive equation. Moreover, based on the work of Lin et al. [35], the Hensel–Spittel equation may have a higher prediction accuracy for describing the hot compressed deformation behavior of TC4–DT alloy, which is also probably achieved by revising the Hensel–Spittel equation.

In present study, to predict the hot compressed flow stress of TC4–DT alloy, the new modified strain-compensated Arrhenius-type (ms–cA-type) and modified Hensel–Spittel (mHS) equation is developed by modifying the strain rate. Moreover, the two new modified constitutive equations are established by combining regression analysis with iterative methods, which is used instead of the traditional linear regression method. Meanwhile, both the original strain-compensated Arrhenius-type (os–cA-type) and original Hensel–Spittel (oHS) equation are established by the traditional method.

A comparative study has been made on the above four constitutive equations, which is used to prove the two modified constitutive equations are more accurate for prediction of the flow stress of TC4–DT alloy.

## 2. Materials and Methods

The flow stress data of TC4–DT alloy at elevated temperature is taken from the literature (Peng et al. [32]), which is shown in Table A1. The chemical composition (in wt%) of the titanium alloy is 6.20Al–4.1V–0.04Fe–0.017C–0.11O–(bal.) Ti. The microstructure is entirely homogeneous equiaxed *α* + *β*, consisting of 70% primary *α* phase with the average grain size 9 μm and transformed *β* with the secondary lamellar a thickness of 1.4 μm.

Cylindrical specimens with 12 mm in height and with 10 mm in diameter are prepared and their flat ends are recessed to a depth of 0.1 mm entrap the lubricant of graphite mixed with machine oil, which can minimize the frictions between the specimens and die. The isothermal hot compression tests were performed at temperatures of 1203, 1218, 1233, 1248, 1263, 1278 and 1293 K with strain rates of 0.001, 0.01, 0.1, 1 and 10 s^−1^. The isothermal hot compression tests of the titanium alloy were conducted on a Gleeble-1500 thermo-simulation system (Dynamic Systems Inc., Poestenkill, NY, USA). The specimen was heated to the deformation temperature at a rate of 10 K/s and held for 3 min to eliminate the thermal gradients.

## 3. Results

To predict the flow stress of TC4–DT titanium alloy, Peng et al. [32] determined two sets of material constants in the original strain-compensated Arrhenius-type equations based on different phase regions, and they modified the original constitutive equation under partial deformation condition. These results mean that the flow stress curves of TC4–DT titanium alloy obtained by Peng et al. [32] are more complicated. Therefore, the experimental data collected by Peng et al. [32] is used in the current study, which is used to prove a stronger predictive ability of the new modified constitutive equations and the authenticity of results.

**Figure 1:**
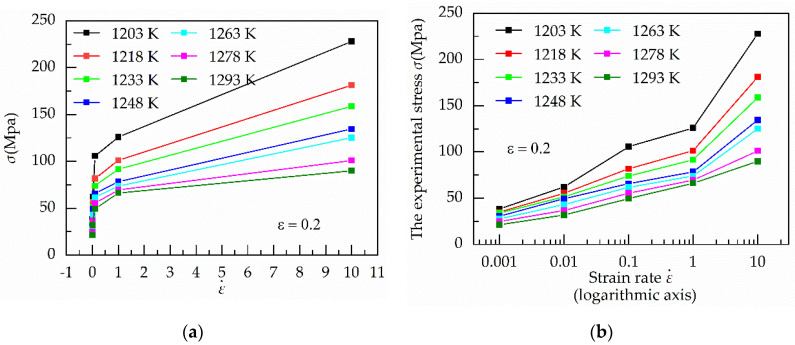
Relationship between (**a**) $\dot{\varepsilon }$ vs. the mean of *σ*; (**b**) $\dot{\varepsilon }$ vs. *σ* under the strain of 0.20 and various deformation temperatures.

**Figure 2:**
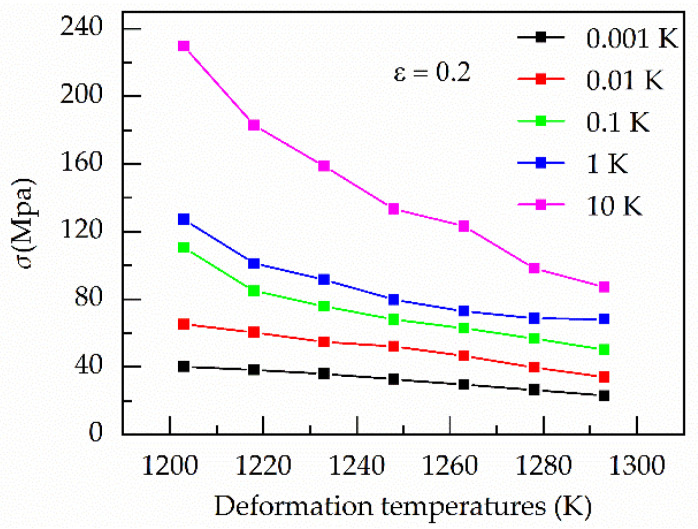
Relationship between deformation temperatures and stress under the strain of 0.20 and various strain rates.

Figure 1 shows the relationship between $\dot{\varepsilon }$ vs. the mean of *σ* and $\dot{\varepsilon }$ vs. *σ* under the strain of 0.20 and various temperatures. It is easily found a strong nonlinear relationship between *σ* and $\dot{\varepsilon }$ at various temperatures. Moreover, as the value of strain rate increases from 1 to 10 s^−1^, the stress increases sharply, especially below 1273 K. Figure 2 shows the relationship between temperatures and stress under the strain of 0.20 and various strain rates. When the value of the strain rate is below 1 s^−1^, the change of stress with temperature is relatively stable. However, under the strain of 10 s^−1^, the stress decreases sharply as the temperature increases.

At hot temperature, the flow stress of metals and alloys is dependent on the interaction between them the work hardening and the dynamic softening mechanisms between the work hardening and the dynamic softening mechanisms. As the strain rate increases, the time required to reach the same strain becomes shorter. Moreover, the dynamic softening mechanisms become increasing insufficient, including the dynamic recovery (DRV) and dynamic recrystallization (DRX). Therefore, stress increase with the rise of the strain rate, especially above 1 s^−1^ in Figure 1b. In contrast, the increase in temperature promotes dynamic recovery (DRV) and dynamic recrystallization (DRX). At the higher stain rate, the softening effect of temperature becomes increasing prominent, especially at 1293 K in Figure 2.

In Figure 1 and Figure 2, differences in strain rates lead to different trends in stress. Therefore, these results mean the strain rate has a significant influence on stress, especially under the higher strain rate.

Based on the above result, revising the strain rate is used to improve the accuracy of the constitutive equation. Therefore, the new modified strain-compensated Arrhenius-type (ms–cA-type) equation and the modified Hensel–Spittel (mHS) equation is established. Moreover, it is also established the original strain-compensated Arrhenius-type (os–cA-type) equation and the original Hensel–Spittel (oHS) equation. The four constitutive equations are used to predict the flow stress of TC4–DT titanium alloy under temperatures of 1203–1293 K and strain rates of 0.001–10 s^−1^. The different constitutive equations are compared to prove that the accuracy of the modified equation is higher.

### 3.1. Establishing the Original Strain-Compensated Arrhenius-Type (os–cA-type) Equation

The relationship between deformation parameters at elevated temperature can be expressed by the original Arrhenius-type equation, which is shown in Equation (1). The hyperbolic sine model in the Arrhenius-type equation gives better approximations for the flow stress of the TC4–DT titanium alloy.

Material constants are solved based on experimental data from the isothermal hot compression tests. The strain of 0.30 is taken as an example to introduce the procedures of solving material constants.

Substituting Equations (4) and (5) in Equation (1), respectively gives:

(6) $$\dot{\varepsilon }=A\sigma^{n_{1}}\exp(-Q/(\mathrm{RT}))$$

(7) $$\dot{\varepsilon }=A\exp(\beta \sigma )\exp(-Q/(\mathrm{RT}))$$

Taking the logarithm of both sides of Equations (6) and (7), respectively yields:

(8) $$\ln\dot{\varepsilon }=\ln A+n_{1}\ln\sigma -Q/(\mathrm{RT})$$

(9) $$\ln\dot{\varepsilon }=\ln A+\beta \sigma -Q/(\mathrm{RT})$$

**Figure 3:**
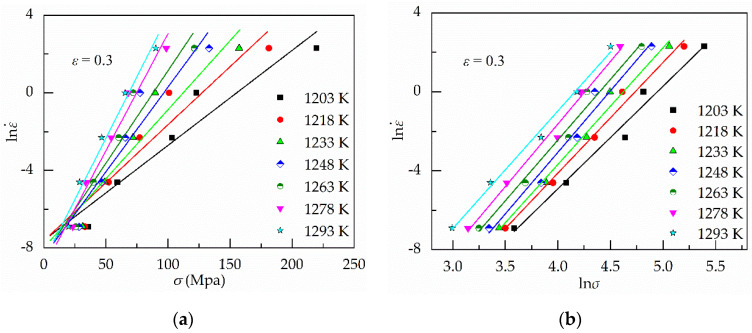
Results of regression analysis for determining *α*. (**a**) Relationship between *σ*–ln$\dot{\varepsilon }$; (**b**) relationship between ln*σ*–ln$\dot{\varepsilon }$.

Then, it is obtained that two groups of straight lines based on Equations (8) and (9), as shown in Figure 3a,b, respectively. The values of *n*_1_ and *β* are the average value of the slope of the fitting lines in the ln*σ*–ln$\dot{\varepsilon }$ and *σ*–ln$\dot{\varepsilon }$ plots, which were 5.9160 and 0.0871, respectively. Then, the value of *α* is 0.0147 based on *α* = *β*/*n*_1_. Moreover, the values of *ασ* varied from 0.29 to 3.23 under the strain of 0.30. Therefore, it is reasonable to choose the constitutive equation under all the strain levels.

For all the strain levels, Equation (1) can be written as:

(10) $$\dot{\varepsilon }=A{\left[\sinh(\alpha \sigma )\right]}^{n}\exp(-Q/(\mathrm{RT}))$$

Taking the logarithm of both sides of Equation (10) gives:

(11) $$\ln\dot{\varepsilon }=n\ln[\sinh(\alpha \sigma )]+\ln A-Q/(\mathrm{RT})$$

**Figure 4:**
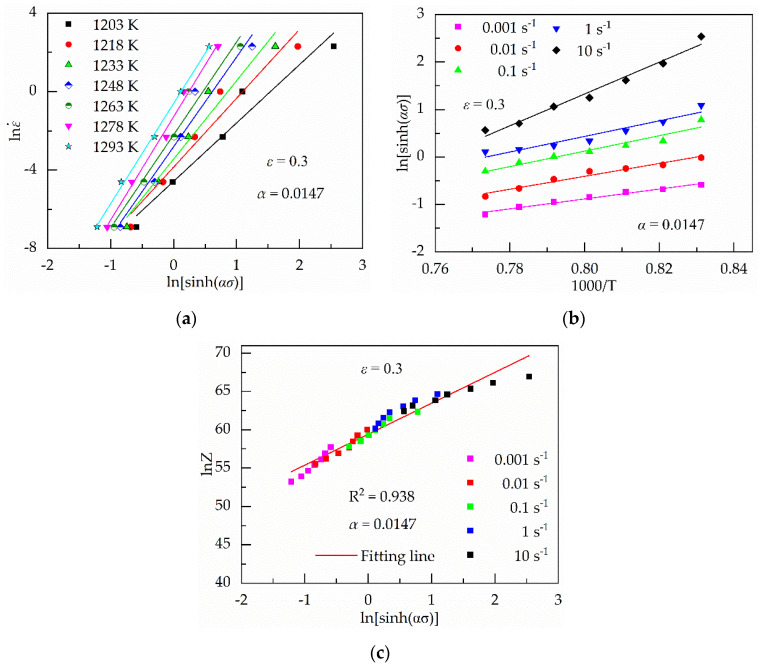
Results of regression analysis based on α = 0.0147. (**a**) Relationship between ln[sinh(*ασ*)]–ln$\dot{\varepsilon }$; (**b**) relationship between 1000/T–ln[sinh(*ασ*)]; (**c**) relationship between ln[sinh(*ασ*)]–lnZ.

The value of *n* can be obtained from the average slope of the lines in ln$\dot{\varepsilon }$–ln[sinh(*ασ*)] plots, as shown in Figure 4a.

Because the value of ln[sinh(*ασ*)] is approximately 1000 times the value of 1/T, 1000/T is used instead of 1/T to obtain a more accurate result. When 1000/T is used, the unit of Q becomes kJ·mol^−1^.

For a particular strain rate, Equation (12) can be derived based on Equation (11), as follows:

(12) $$Q=Rn\frac{\partial \ln[\sin h(\alpha \sigma )]}{\partial 1000/T}$$

Therefore, the value of Q can be gained from the average slope of the lines in ln[sinh(*ασ*)]–1000/T under various strain rates, as shown in Figure 4b.

Substituting Equation (2) in Equation (10) and taking the logarithm of both sides of Equation (10) give:

(13) lnZ=lnA+n[ln(sinh(ασ))]

Based on Equation (13), it is easily found that the relationship between ln[sinh(*ασ*)] and ln*Z* is linear. Moreover, the value of lnA is the ln*Z*-intercept of the fitting lines in the ln[sinh(*ασ*)]–ln*Z* plots when the value of ln[sinh(*ασ*)] is zero, as shown in Figure 4c. The values of *n*, Q and lnA are 4.31, 646.50 and 59.43, respectively.

**Figure 5:**
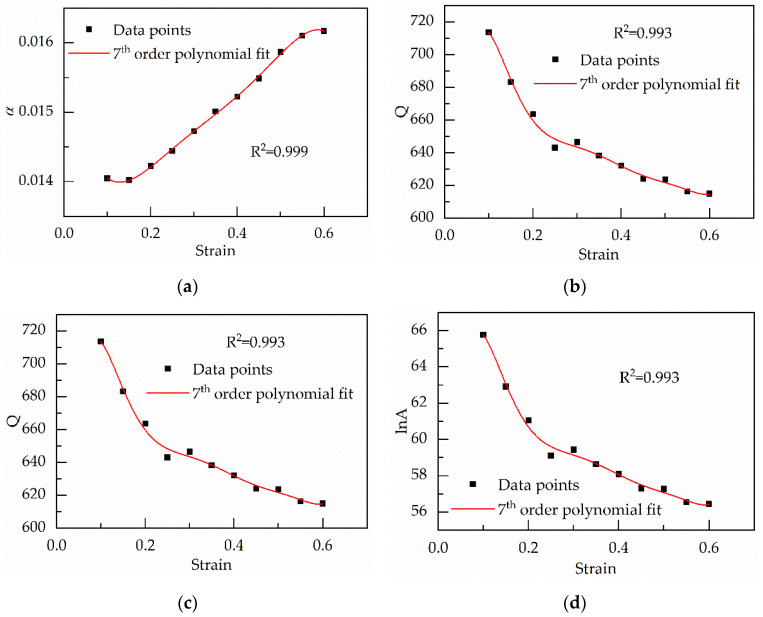
Variation of (**a**) *α*; (**b**) *n*; (**c**) lnA; (**d**) Q with true strain represented by a 7th order of the polynomial.

In summary, the values of material constants *α*, *n*, Q and lnA are obtained under a single strain, namely *ε* = 0.30. However, when the strain is different, the values of the material constants will change [21]. The constitutive equation is established based on strain range from 0.10 to 0.60 at an interval of 0.05. The above solving process is repeated to obtain material constants under various strains. By adjusting the order of the polynomial, it is found that a seventh order of the polynomial is employed to represent the influence of strain on material constants with a very good correlation and generalization, which is shown in Figure 5 and Equation (14) [35]. Moreover, the values of multinomial coefficients are shown in Table 1.

(14) $$\left\{ \begin{array}{l} \alpha =a_{0}+a_{1}\varepsilon +a_{2}\varepsilon^{2}+a_{3}\varepsilon^{3}+a_{4}\varepsilon^{4}+a_{5}\varepsilon^{5}+a_{6}\varepsilon^{6}+a_{7}\varepsilon^{7}\\ n=N_{0}+N_{1}\varepsilon +N_{2}\varepsilon^{2}+N_{3}\varepsilon^{3}+N_{4}\varepsilon^{4}+N_{5}\varepsilon^{5}+N_{6}\varepsilon^{6}+N_{7}\varepsilon^{7}\\ Q=Q_{0}+Q_{1}\varepsilon +Q_{2}\varepsilon^{2}+Q_{3}\varepsilon^{3}+Q_{4}\varepsilon^{4}+Q_{5}\varepsilon^{5}+Q_{6}\varepsilon^{6}+Q_{7}\varepsilon^{7}\\ \ln A=A_{0}+A_{1}\varepsilon +A_{2}\varepsilon^{2}+A_{3}\varepsilon^{3}+A_{4}\varepsilon^{4}+A_{5}\varepsilon^{5}+A_{6}\varepsilon^{6}+A_{7}\varepsilon^{7} \end{array}\right.$$

When the materials constant can be evaluated based on Equation (14), the flow stress can be predicted from Equation (15) [14,32], which is derived from Equations (1), (2) and (5).

(15) $$\left\{ \begin{array}{l} \sigma =\frac{1}{\alpha }\ln\left\{{\left(\frac{Z}{A}\right)}^{1/n}+{\left[{\left(\frac{Z}{A}\right)}^{2/n}+1\right]}^{1/2}\right\}\\ Z=\dot{\varepsilon }\exp(Q/(\mathrm{RT})) \end{array}\right.$$

### 3.2. Establishing the Modified Strain-Compensated Arrhenius-Type (ms–cA-type) Equation

The modified strain-compensated Arrhenius-type (ms–cA-type) equation is developed by revising the strain rate in the original strain-compensated Arrhenius-type equation. Namely, the constitutive equation is modified by using an effective strain rate ${\dot{\varepsilon }}^{*}$ instead of real strain rate $\dot{\varepsilon }$ again. The modified Arrhenius-type equation is shown in Equation (16).

(16) $${\dot{\varepsilon }}^{*}=A{\left[\sinh(\alpha \sigma )\right]}^{n}\exp(-Q/(\mathrm{RT}))$$

where ${\dot{\varepsilon }}^{*}$ is the effective strain rate (s^−1^), *σ* is the flow stress (MPa), T is the deformation temperature (K), Q is the activation energy (J·mol^−1^), R is the molar gas constant (8.3145 J·mol^−1^·K^−1^), A, *α*, *n* and *m* are material constants.

First, material constants are obtained to establish the modified strain-compensated Arrhenius-type equation. Because material constants A, *α*, *n*, *m* and Q change with strain, it is necessary to solve material constants under various strain. After obtaining material constants under different strain, the effective strain rate can be determined.

#### 3.2.1. Determining Material Constants

Wang et al. [26] proposed material constants in the Arrhenius-type equation were solved by combining iterative methods with regression analysis. In the prevent study, the same method is used to solve the material constants in the modified Arrhenius-type equation. During solving material constants, A, *α*, *n*, *m* and Q, the real strain rate $\dot{\varepsilon }$ is used instead of the effective strain rate ${\dot{\varepsilon }}^{*}$ in Equation (16). Namely, substituting ${\dot{\varepsilon }}^{*}=\dot{\varepsilon }$ in Equation (16) gives:

(17) $$\dot{\varepsilon }=A{\left[\sinh(\alpha \sigma )\right]}^{n}\exp(-Q/(\mathrm{RT}))$$

Taking the logarithm of both sides of Equation (17) gives:

(18) $$\ln\dot{\varepsilon }=\ln A+n\ln[\sinh(\alpha \sigma )]-Q/(\mathrm{RT})$$

Rearranging gives:

(19) $$\sinh^{-1}\{\exp[\ln\dot{\varepsilon }/n+Q/(n\mathrm{RT})-\ln(A)/n]\}=\alpha \sigma$$

where sinh^−1^ is the inverse function of sinh.

1000/T is used instead of 1/T to obtain a more accurate result again. Therefore, the unit of Q becomes kJ·mol^−1^.

Based on Equations (18) and (19), Equations (20) and (21) is established, respectively.

(20) $$\left\{ \begin{array}{l} y_{1}=\ln\dot{\varepsilon }\\ x_{1}=\ln[\sinh(\alpha \sigma )]\\ x_{2}=-1000/(\mathrm{RT})\\ b_{1}=n\\ b_{2}=Q\\ b_{3}=\ln(A) \end{array}\right.$$

(21) $$\left\{ \begin{array}{l} y_{2}=\sinh^{-1}\{\exp[\ln\dot{\varepsilon }/n+1000Q/(n\mathrm{RT})-\ln(A)/n]\}\\ x_{3}=\sigma \\ c=\alpha \end{array}\right.$$

Substituting Equations (20) and (21) in Equations (18) and (19), respectively gives:

(22) $$y_{1}=b_{1}x_{1}+b_{2}x_{2}+b_{3}$$

(23) $$y_{2}=cx_{4}$$

If *α* is determined, the *n*, Q and A (*b*_1_, *b*_2_ and *b*_3_) will be obtained by performing multiple linear regression on Equation (22). Similarly, if *n*, Q and A are determined, the *α* (c) will be obtained by performing linear regression on Equation (23). Therefore, iterative methods can be used to solve material constants.

Based on the above analysis, it is used that a new method combines regression analysis with iterative methods. About the new method, first, the initial guess of *α* is determined to solve *x*_1_ in Equation (23). Second, the approximation of *n*, Q and A is determined by performing multiple linear regression on Equation (22). Third, *n*, Q and A obtained in the second step are substituted in Equation (21) to determine *y*_2_, and then the approximation of *α* is determined by performing linear regression on Equation (23). Fourthly, the approximation of *α* from one iteration is the input of the next to iterate Equation (22) and (23) until a stopping criterion is met. Finally, it is obtained the convergence values of *n*, Q, A and *α* under a single strain.

The above four steps are repeated for solving material constants under different strain. When the stopping criterion is established based on the predicted stress under all strain, it is used to obtain the convergence values of *n*, Q, A and *α* under various strain. The solution process under a single strain is as follows:

The strain of 0.30 is still taken as an example to introduce the procedures of solving material constants *n*, Q, A and *α* under a single strain. The real strain rate is used to replace the effective strain rate during determining material constants.

At first, the initial guess of *α* is the value of *α* in the original Arrhenius-type equation under strain of 0.30. Namely, the initial guess of *α* is equal to 0.0147. Next, it is needed to determine the value of *x*_1_. At a special strain, there are seven different deformation temperatures (1203, 1218, 1233, 1248, 1263, 1278 and 1293 K) and five different values of strain (0.001, 0.01, 0.1, 1 and 10 s^−1^), which can be obtained based on the selected experimental data points. Namely, there are thirty-five experimental data points at a special strain. The set of *x*_1_-values can be obtained based on the experimental data points and Equation (20). The multiple linear regression is performed based on Equation (22), where *x*_1_, *x*_2_ and *x*_3_ are the independent variables, *y*_1_ is the dependent variable and *b*_1_, *b*_2_ and *b*_3_ are the unknown parameters.

Newton’s method is used to perform multiple linear regression. For Newton’s method, the initial value of the unknown parameters is needed. The initial guess of *b*_1_, *b*_2_ and *b*_3_ are 4.31, 646.50 and 59.43, respectively. After finishing multiple linear regression, it is obtained that the approximate value of *b*_1_, *b*_2_ and *b*_3_ are 3.99, 597.50 and 54.72, respectively.

Then, the set of *y*_2_-values is obtained by substituting the value of *b*_1_, *b*_2_ and *b*_3_ in Equation (21). Linear regression can be performed based on Equation (23). During linear regression, Equation (23) is the regression model, *x*_4_ is the independent parameters, *y*_1_ is the dependent variable and *c* is the unknown variables. Newton’s method is still used to perform linear regression and the initial guess of *α* is still equal to 0.0147. After finishing linear regression, it is obtained that the approximate value of *c* (*α*) is 0.0146. To date, the approximate values of *n*, Q, A and *α* are obtained. Namely, it is obtained the results of the first iteration, which is shown in Equation (24).

(24) $$\left\{ \begin{array}{l} \sigma =\frac{1}{0.0146}\ln\left\{{\left(\frac{Z}{54.72}\right)}^{1/3.99}+{\left[{\left(\frac{Z}{54.72}\right)}^{2/3.99}+1\right]}^{1/2}\right\}\\ Z=\dot{\varepsilon }\exp(597.50\times 10^{3}/\mathrm{RT}) \end{array}\right.(1\mathrm{th}\;\mathrm{iteration})$$

The approximate value of *α* is the input of the next iteration to continue to iterate Equations (22) and (23). After the 253rd iteration, the approximate values of *b*_1_, *b*_2_, *b*_3_ and *c* are 5.86, 586.27, 80.10 and 1.927 × 10^−4^, respectively. Therefore, a new result is obtained, which is shown in Equation (25). The strain rate is not modified in Equations (24) and (25).

(25) $$\left\{ \begin{array}{l} \sigma =\frac{1}{1.927\times 10^{-4}}\ln\left\{{\left(\frac{Z}{80.10}\right)}^{1/5.86}+{\left[{\left(\frac{Z}{80.10}\right)}^{2/5.86}+1\right]}^{1/2}\right\}\\ Z=\dot{\varepsilon }\exp(586.27\times 10^{3}/\mathrm{RT}) \end{array}\right.(253\mathrm{th}\;\mathrm{iteration})$$

MATLAB software is used for programming and the *nlinfit* function is called to conduct regression analysis based on Newton’s method.

**Figure 6:**
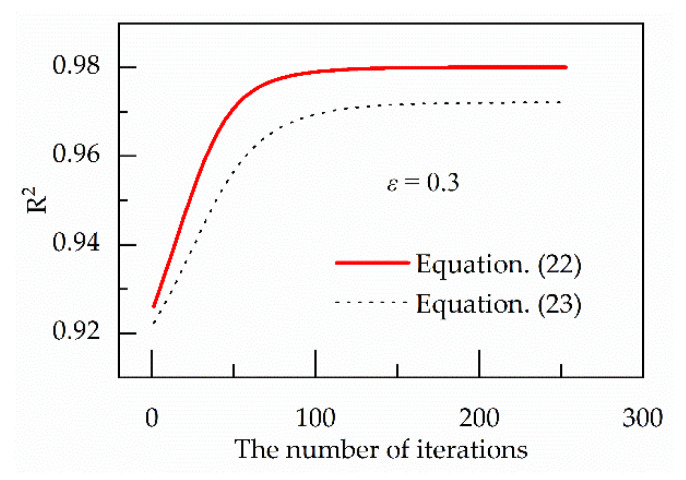
Variation of R^2^ of Equations (22) and (23) with iterations.

In the above iterative process, the same number of data points and the same regression model are used in regression analysis and the prediction accuracy can be verified by the coefficient of determination (R^2^), which is shown in Equation (26) [36]. The higher the R^2^-value, the higher the prediction accuracy. Figure 6 shows R^2^-values of Equations (22) and (23) keep increasing with increasing the number of iterations, which means the prediction accuracy is continuously improved with increasing the number of iterations. Therefore, the prediction accuracy of Equation (25) is higher.

(26) $$R^{2}=1-\frac{\sum_{i=1}^{N}{\left(E_{i}-P_{i}\right)}^{2}}{\sum_{i=1}^{N}{\left(E_{i}-\bar{E}\right)}^{2}}$$

where *E_i_* is the experimental data, *P_i_* is the predicted value obtained from the constitutive equation, $\bar{E}$ is the mean value of *E_i_* and *N* is the total number of data employed in the investigation.

To date, it is shown the process of solving the material constants under a single strain. Then the stopping criterion is determined to obtain material constants in the strain range from 0.10 to 0.60 at an interval of 0.05.

About the stopping criterion, average absolute relative error (AARE) is an unbiased statistical parameter that is calculated via a term-by-term comparison of the relative error [12], as shown in Equation (27). Moreover, AARE is always used to verify the accuracy of the constitutive models. Therefore, AARE-value is obtained based on the predicated stress in the strain range from 0.10 to 0.60 at an interval of 0.05. Moreover, the stopping criterion is the error of AARE from the previous iteration to this one, as shown in Equation (28).

(27) $$\mathrm{AARE}(\%)=\frac{1}{N}\sum_{i=1}^{N_{i}}\frac{\left|E_{i}-P_{i}\right|}{E_{i}}\times 100$$

where *E_i_* is the experimental data, *P_i_* is the predicted value obtained from the constitutive equation and *N* is the total number of data points.

(28) $${\mathrm{AARE}}_{i}-{\mathrm{AARE}}_{i-1}\leq 10^{-4}$$

where AARE*_i_* and AARE*_i_*_−1_ is the average absolute relative error from the *i*th and *i* − 1th iteration.

**Figure 7:**
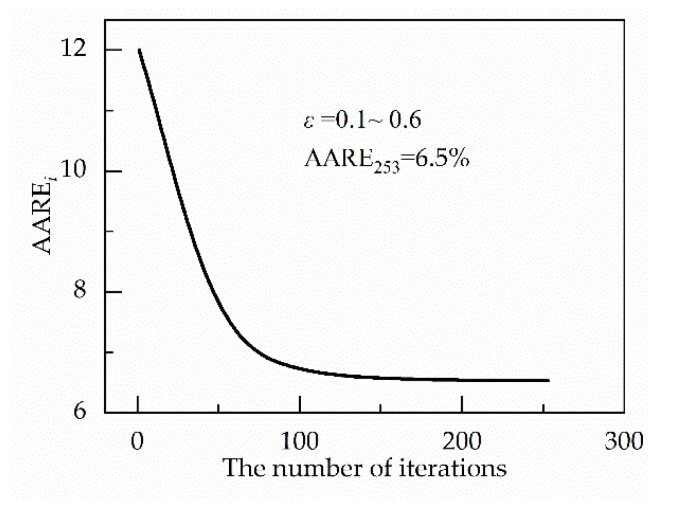
Variation of average absolute relative error (AARE)*_i_* with iterations.

**Figure 8:**
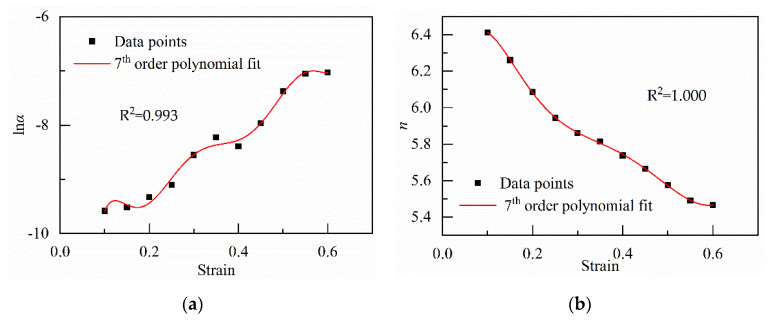

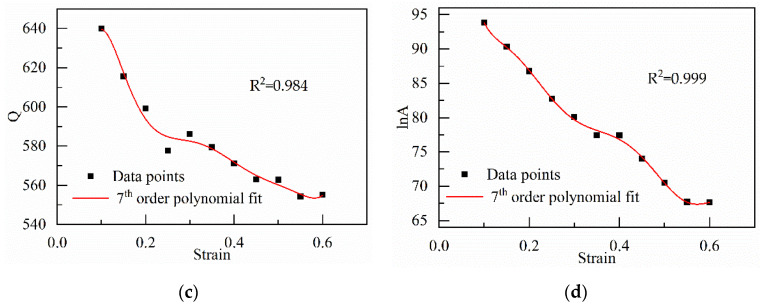
Variation of (**a**) ln*α*; (**b**) *n*; (**c**) ln A; (**d**) Q with true strain represented by a 7th order of the polynomial.

The value of AARE*_i_* changes with the number of iterations, as shown in Figure 7. After the 253rd iteration, the stopping criterion is met. AARE_235_ is equal to 6.53%. It is also obtained that material constants under strain in the range from 0.10 to 0.60. In addition, a seventh-order polynomial is used to describe the relationship between material constants and strain, which is shown in Figure 8. The coefficients of the polynomial functions are given in Table 2. Because the values of *α* are far less than 10^−3^, the logarithm of *α* is used to describe the correlation between the strain and it.

#### 3.2.2. Determining Effective Strain Rate

By the above process, it is obtained the value of *n*, Q, A and *α* under the strain range from 0.10 to 0.60 at an interval of 0.05. After that, the effective strain rate ${\dot{\varepsilon }}^{*}$ is solved. The process of solving ${\dot{\varepsilon }}^{*}$ is as follows:

At a special strain rate, there are seven different deformation temperatures (1203, 1218, 1233, 1248, 1263, 1278 and 1293 K) and eleven different values of strain (0.10, 0.15, 0.20, 0.25, 0.30, 0.35, 0.40, 0.45, 0.50, 0.55 and 0.60), which can be obtained based on the selected experimental data points. Namely, there are seventy-seven experimental data points at a special strain rate. The values of strain, stress, and deformation temperatures in an experimental data point are substituted in Equation (17). Moreover, it is obtained a value of strain rate. Finally, based on the seventy-seven experimental data points, the seventy-seven values of strain rates can be obtained. Because there are errors in the process of solving the material parameters, there are differences between the seventy-seven values of strain rates. Based on least squares, the mean of the seventy-seven values of strain rates is used as the effective strain rate ${\dot{\varepsilon }}^{*}$ at the special strain rate, which is shown in Equation (29).

(29) $${\dot{\varepsilon }}^{*}=\frac{1}{N}\sum_{\varepsilon =0.1}^{0.6}\sum_{T=1203K}^{1293K}\;A_{\varepsilon }^{\prime }{\left[\sinh(\alpha_{\varepsilon }\sigma_{\varepsilon ,T})\right]}^{n_{\varepsilon }^{\prime }}\exp(-Q_{\varepsilon }^{\prime }/(\mathrm{RT}))$$

where ${\dot{\varepsilon }}^{*}$ is the effective strain rate under a special strain rate; *ε* is the strain; *n_ε_*, Q*_ε_*, A*_ε_* and *α*_ε_ are the corresponding material constants under the strain *ε*; T is the deforming temperature; *σ*_ε,T_ is the corresponding experimental stress under the strain *ε*, the temperature T and the special strain rate; and *N* is the total number of data points under the special strain rate.

**Figure 9:**
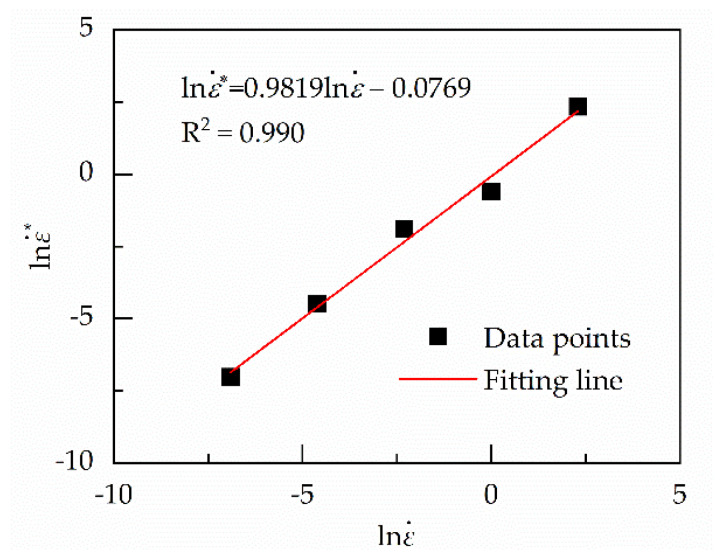
Relation between ln${\dot{\varepsilon }}^{*}$ and ln$\dot{\varepsilon }$.

The corresponding ${\dot{\varepsilon }}^{*}$ at different $\dot{\varepsilon }$ can be obtained by repeating the above process. Because the maximum of the real and effective strain rate is ten times more than the minimum values, the logarithm of ${\dot{\varepsilon }}^{*}$ and $\dot{\varepsilon }$ is used to describe their correlation, which is shown in Equation (30) and Figure 9. The coefficients of the linear functions are given in Table 2.

(30) $$\ln{\dot{\varepsilon }}^{*}=E_{0}+E_{1}\ln\dot{\varepsilon }$$

**Figure 10:**
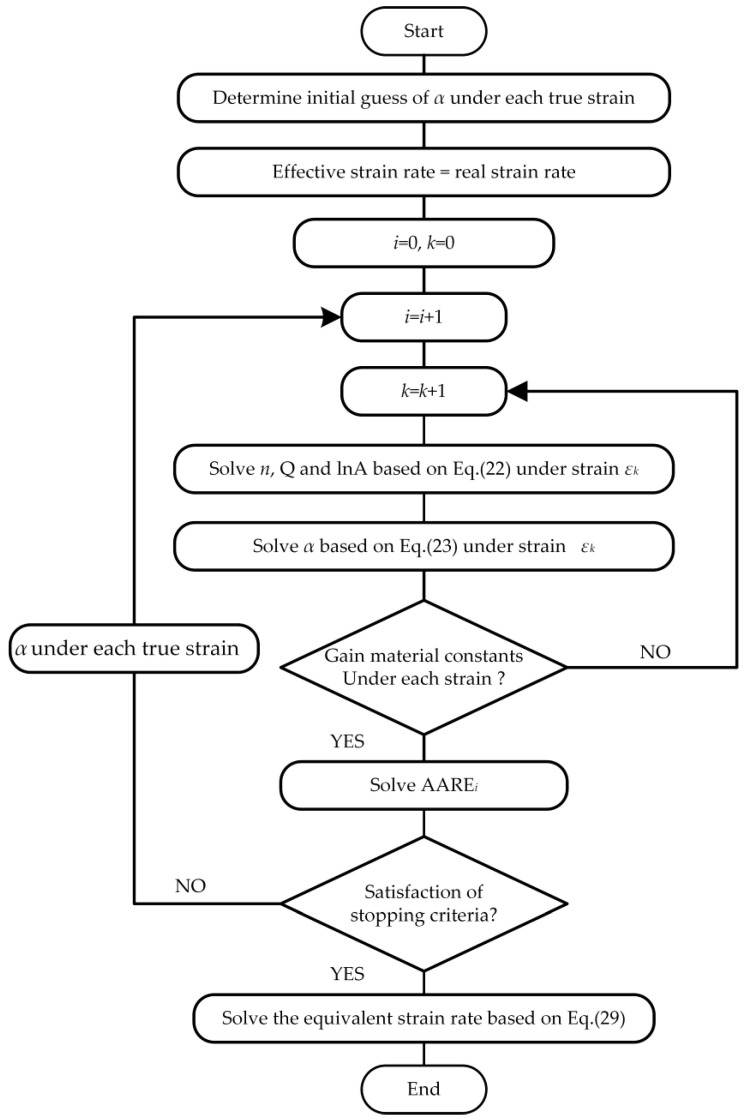
Flow chart of establishing the modified strain-compensated Arrhenius-type equation.

In summary, material constants and effective strain rate are determined based on the above solution procedure, which is shown in Figure 10. The modified Arrhenius-type equation is derived from Equations (16) and (30), as shown in Equation (31).

(31) $$\exp(E_{0}+E_{1}\ln\dot{\varepsilon })=A{\left[\sinh(\alpha \sigma )\right]}^{n}\exp(-Q/(\mathrm{RT}))$$

To simplify form of Equation (31), Z* is used instead of *Z* in Equation (2) While ${\dot{\varepsilon }}^{*}$ in Equation (30) is used instead of $\dot{\varepsilon }$ in Equation (2). Therefore, a new equation is developed, as follows:

(32) $$Z^{*}=\exp(E_{0}+E_{1}\ln\dot{\varepsilon }+Q/(\mathrm{RT}))$$

Substituting Equation (32) in Equation (31) and rearranging give:

(33) $$\left\{ \begin{array}{l} \sigma =\frac{1}{\alpha }\ln\left\{{\left(\frac{Z^{*}}{A}\right)}^{1/n}+{\left[{\left(\frac{Z^{*}}{A}\right)}^{2/n}+1\right]}^{1/2}\right\}\\ Z^{*}=\exp(E_{0}+E_{1}\ln\dot{\varepsilon }+Q/(RT)) \end{array}\right.$$

**Figure 11:**
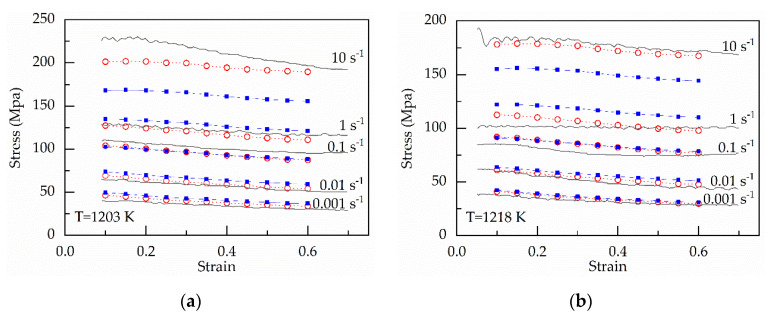

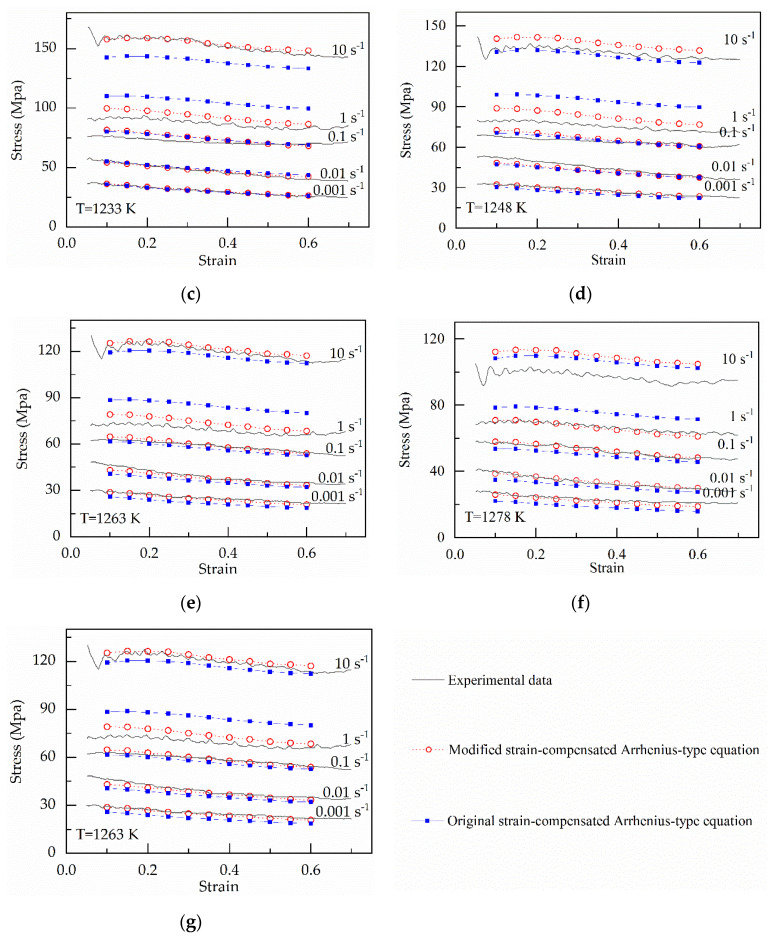
Comparison between the experimental and predicted stress by the original and modified strain-compensated Arrhenius-type equations at (**a**) 1203 K; (**b**) 1218 K; (**c**) 1233 K; (**d**) 1248 K; (**e**) 1263 K; (**f**) 1278 K; (**g**) 1293 K.

When the parameters in Equation (33) can be evaluated based on Equation (34) and Table 2, the flow stress can be predicted, which is shown in Figure 11.

(34) $$\left\{ \begin{array}{l} \ln\alpha =a_{0}+a_{1}\varepsilon +a_{2}\varepsilon^{2}+a_{3}\varepsilon^{3}+a_{4}\varepsilon^{4}+a_{5}\varepsilon^{5}+a_{6}\varepsilon^{6}+a_{7}\varepsilon^{7}\\ n=N_{0}+N_{1}\varepsilon +N_{2}\varepsilon^{2}+N_{3}\varepsilon^{3}+N_{4}\varepsilon^{4}+N_{5}\varepsilon^{5}+N_{6}\varepsilon^{6}+N_{7}\varepsilon^{7}\\ Q=Q_{0}+Q_{1}\varepsilon +Q_{2}\varepsilon^{2}+Q_{3}\varepsilon^{3}+Q_{4}\varepsilon^{4}+Q_{5}\varepsilon^{5}+Q_{6}\varepsilon^{6}+Q_{7}\varepsilon^{7}\\ \ln A=A_{0}+A_{1}\varepsilon +A_{2}\varepsilon^{2}+A_{3}\varepsilon^{3}+A_{4}\varepsilon^{4}+A_{5}\varepsilon^{5}+A_{6}\varepsilon^{6}+A_{7}\varepsilon^{7} \end{array}\right.$$

### 3.3. Establishing the Original Hensel–Spittel (oHS) Equation

Hensel and Spittel proposed a constitutive equation (namely HS equation) to predict the flow curves of alloy [27], which is shown in Equation (35).

(35) $$\sinh(\alpha \sigma )=A\exp(m_{1}T)\varepsilon^{m_{2}}{\dot{\varepsilon }}^{m_{3}}\exp(m_{4}/\varepsilon ){\left(1+\varepsilon \right)}^{m_{5}T}\exp(m_{6}\varepsilon ){\dot{\varepsilon }}^{m_{7}T}T^{m_{8}}$$

where *α*, A, *m*_1_, *m*_2_, *m*_3_, *m*_4_, *m*_5_, *m*_6_, *m*_7_ *and m*_8_ are material parameters. Generally, *m*_7_ and *m*_8_ are neglected.

Taking the logarithms on both sides of Equation (35) gives:

(36) $$\ln[\sinh(\alpha \sigma )]=\ln(A)+m_{1}T+m_{2}\ln(\varepsilon )+m_{3}\ln(\dot{\varepsilon })+m_{4}/\varepsilon +m_{5}T\ln(1+\varepsilon )+m_{6}\varepsilon$$

About Equation (36), *α* is a function of stain. The values of *α* can be gained based on the seventh polynomial related to strain, which is shown in Figure 5a [37]. Other material parameters can be obtained by multiple linear regression, which is listed in Table 3.

### 3.4. Establishing the Modified Hensel–Spittel (mHS) Equation

In the present study, the modified Hensel–Spittel (mHS) equation is established by using the effective strain rate ${\dot{\varepsilon }}^{*}$ instead of the real strain rate $\dot{\varepsilon }$, which is shown in Equation (37).

(37) $$\sinh(\alpha \sigma )=A\exp(m_{1}T)\varepsilon^{m_{2}}{\left({\dot{\varepsilon }}^{*}\right)}^{m_{3}}\exp(m_{4}/\varepsilon ){\left(1+\varepsilon \right)}^{m_{5}T}\exp(m_{6}\varepsilon ){\dot{\varepsilon }}^{m_{7}T}T^{m_{8}}$$

where the *α*, A, *m*_1_, *m*_2_, *m*_3_, *m*_4_, *m*_5_, *m*_6_, *m*_7_ and *m*_8_ are material parameters, *α* is a fixed value independent of strain, ${\dot{\varepsilon }}^{*}$ is a function of $\dot{\varepsilon }$.

About solving material parameters in mHS equation, there is a process similar to solving the parameters in the modified strain-compensated Arrhenius-type equation. At first, the real strain rate $\dot{\varepsilon }$ is used instead of the effective strain rate ${\dot{\varepsilon }}^{*}$ in Equation (37). Namely, the modified Hensel–Spittel (mHS) equation is transformed into Equation (35). Next, the material parameters can be solved by combining regression analysis with iterative methods. Because it is needed to perform regression analysis, Equation (35) is transformed into Equation (36).

Rearranging Equation (36) gives:

(38) $$\mathrm{asinh}[\exp(\ln(A)+m_{1}T+m_{2}\ln(\varepsilon )+m_{3}\ln(\dot{\varepsilon }*)+m_{4}/\varepsilon +m_{5}T\ln(1+\varepsilon )+m_{6}\varepsilon )]=\alpha \sigma$$

Based on Equations (36) and (38), Equations (39) and (40) are established, respectively.

(39) $$\left\{ \begin{array}{l} y_{1}=\ln[\sinh(\alpha \sigma )]\\ x_{1}=T\\ x_{2}=\ln(\varepsilon )\\ x_{3}=\ln(\dot{\varepsilon })\\ x_{4}=1/\varepsilon \\ x_{5}=T\ln(1+\varepsilon )\\ x_{6}=\varepsilon \\ b_{i}=m_{i}(i=1,2,3,4,5,6)\\ b_{7}=\ln(A) \end{array}\right.$$

(40) $$\left\{ \begin{array}{l} y_{2}=\mathrm{asinh}[\exp(\ln(A)+m_{1}T+m_{2}\ln(\varepsilon )+m_{3}\ln({\dot{\varepsilon }}^{*})+m_{4}/\varepsilon +m_{5}T\ln(1+\varepsilon )+m_{6}\varepsilon )]\\ x_{7}=\sigma \\ c=\alpha \end{array}\right.$$

Substituting Equations (39) and (40) in Equations (36) and (38), respectively gives:

(41) $$y_{1}=b_{1}x_{1}+b_{2}x_{2}+b_{3}x_{3}+b_{4}x_{4}+b_{5}x_{5}+b_{6}x_{6}+b_{7}$$

(42) $$y_{2}=cx_{7}$$

**Figure 12:**
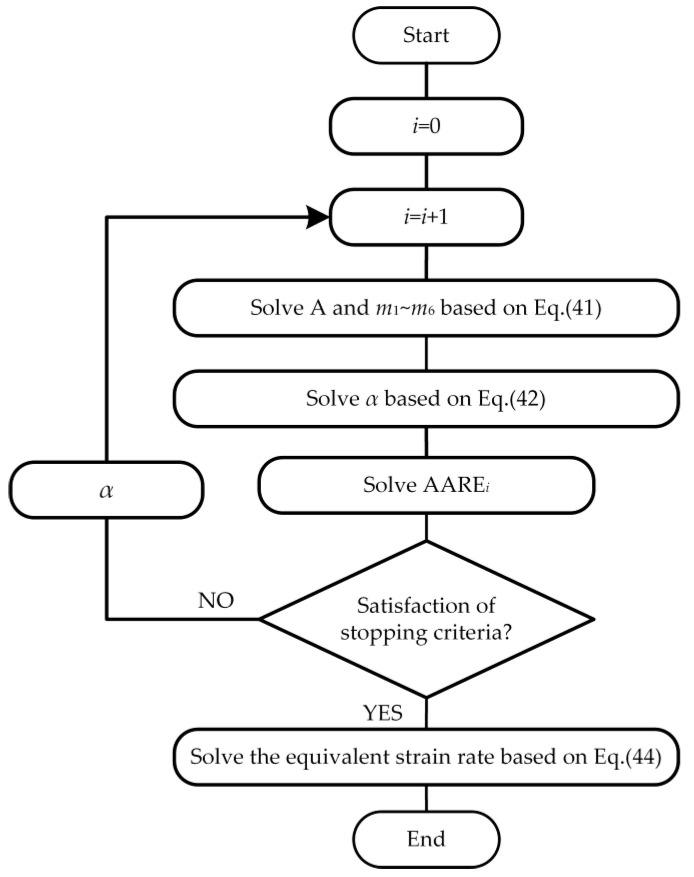
Flow chart of establishing the modified Hensel–Spittel (mHS) equation.

In summary, Equation (37) is transformed into two linear equations, namely Equation (41) and Equation (42). If the material parameter *α* can be determined, the other material parameters will be obtained by performing multiple regression analysis based on Equation (41). Similarly, if the parameters *m*_1_~*m*_6_ can be determined, the material parameter *α* will be obtained by performing regression analysis based on Equation (42). A new iterative process is established based on the above analysis, which is shown in Figure 12. The material parameters can be solved by combining regression analysis with iterative methods. The initial guess of *α* is 0.0147 for iterative methods. The stopping criterion is still Equation (28). When the stopping criterion is met, the material parameters *m*_1_~*m*_6_, *α*, and A can be gained, which is shown in Table 4.

After that, the effective strain rates can be solved based on the material parameters. At first, the real strain rate is separated from the constitutive equation. Rearranging Equation (35) gives:

(43) $$\dot{\varepsilon }={\left[\frac{\sinh(\alpha \sigma )}{A\;\exp(m_{1}T)\varepsilon^{m_{2}}\exp(m_{4}/\varepsilon ){\left(1+\varepsilon \right)}^{m_{5}T}\exp(m_{6}\varepsilon ){\dot{\varepsilon }}^{m_{7}T}T^{m_{8}}}\right]}^{1/m_{3}}$$

Next, based on least squares, it is solved the optimal of the strain rate, which is used as the effective strain rate. At a special strain rate, there are seventy-seven experimental data points. The values of strain, stress, and deformation temperatures in experimental data points are substituted in Equation (43). As a result, it is obtained the seventy-seven values of strain rates. Based on least squares, the mean of the seventy-seven values of strain rates is used as the effective strain rate at the special strain rate ${\dot{\varepsilon }}^{*}$, as follows:

(44) $${\dot{\varepsilon }}^{*}=\frac{1}{N}\sum_{\varepsilon =0.1}^{0.6}\sum_{T=1203K}^{1293K}{\left[\frac{\sinh(\alpha \sigma_{{\dot{\varepsilon }}_{0}})}{A\exp(m_{1}T)\varepsilon^{m_{2}}\exp(m_{4}/\varepsilon ){\left(1+\varepsilon \right)}^{m_{5}T}\exp(m_{6}\varepsilon )}\right]}^{1/m_{3}}$$

where the *α*, A, *m*_1_, *m*_2_, *m*_3_, *m*_4_, *m*_5_ and *m*_6_ are material parameters, T is temperature, ε is the strain, $\sigma_{{\dot{\varepsilon }}_{0}}$ is stress under T, ε and a special strain rate ${\dot{\varepsilon }}_{0}$, *N* is the total number of data employed in the investigation, and ${\dot{\varepsilon }}^{*}$ is the corresponding the effective strain rate at a special strain rate ${\dot{\varepsilon }}_{0}$.

**Figure 13:**
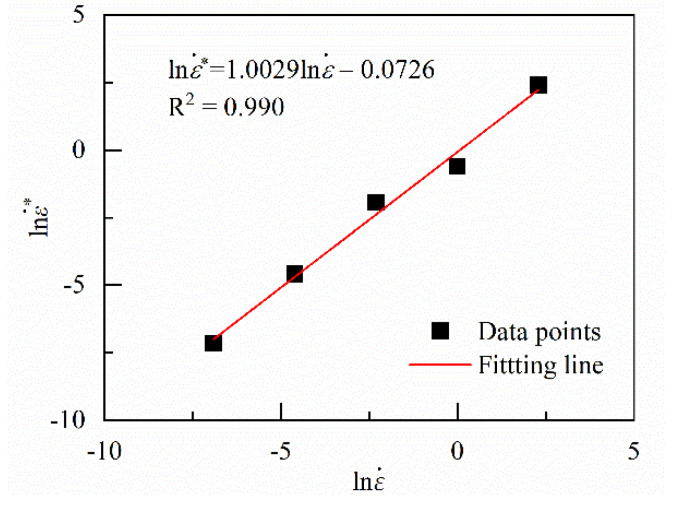
Relation between ln${\dot{\varepsilon }}^{*}$ and ln$\dot{\varepsilon }$ in the modified Hensel–Spittel (mHS) equation.

The corresponding ${\dot{\varepsilon }}^{*}$ at different $\dot{\varepsilon }$ can be obtained by repeating the above process. Then, it is established a relationship between the strain rate and the effective strain rate. The linear equation can be used to describe the relationship between ${\dot{\varepsilon }}^{*}$ and $\dot{\varepsilon }$, which is shown in Equation (45) and Figure 13. In Equation (45), the values of *e*_0_ and *e*_1_ are 0.1668 and −0.0863, respectively.

(45) $$\ln{\dot{\varepsilon }}^{*}=e_{0}+e_{1}\ln\dot{\varepsilon }$$

Rearranging Equation (45) gives:

(46) $${\dot{\varepsilon }}^{*}=e+{\dot{\varepsilon }}^{e_{1}}$$

where *e* = exp(*e*_0_).

Finally, the new modified constitutive equation is developed by using the effective strain rate instead of the strain rate in the original constitutive equation. Substituting Equation (46) in Equation (37) gives:

(47) $$\sinh(\alpha \sigma )=A\exp(m_{1}T)\varepsilon^{m_{2}}{\left(e+{\dot{\varepsilon }}^{e_{1}}\right)}^{m_{3}}\exp(m_{4}/\varepsilon ){\left(1+\varepsilon \right)}^{m_{5}T}\exp(m_{6}\varepsilon ){\dot{\varepsilon }}^{m_{7}T}T^{m_{8}}$$

**Figure 14:**
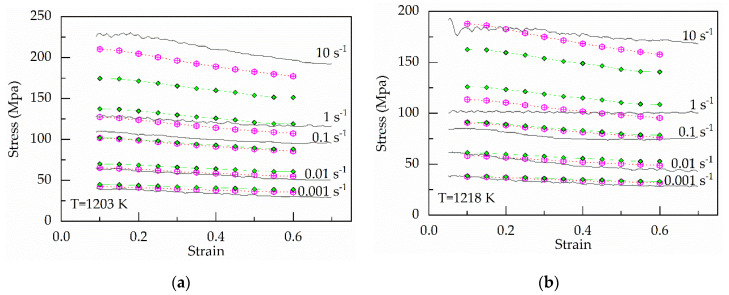

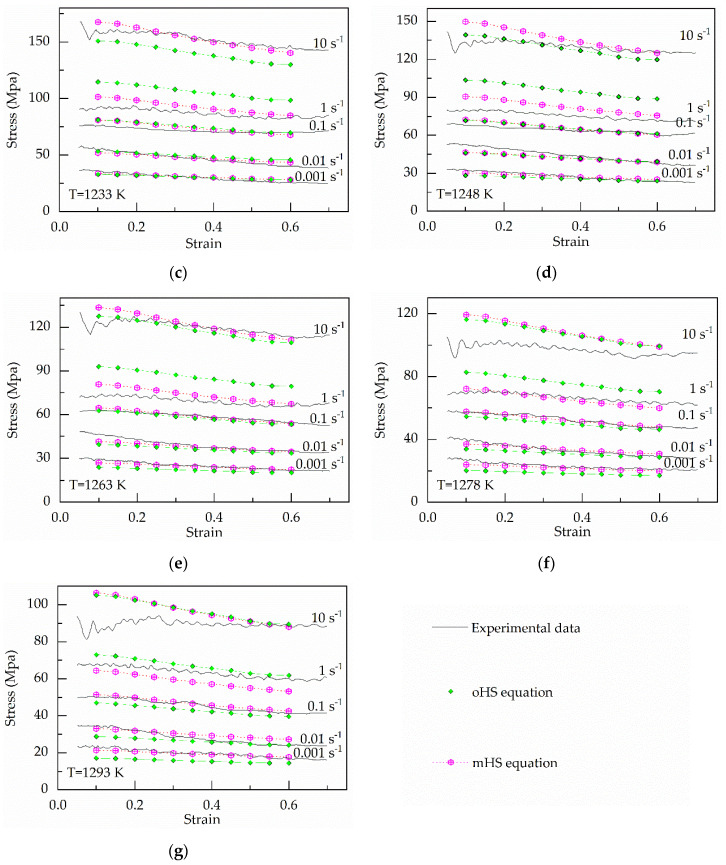
Comparison between the experimental and predicted stress from the original Hensel–Spittel (oHS) equation and the modified Hensel–Spittel (mHS) equation at (**a**) 1203 K; (**b**) 1218 K; (**c**) 1233 K; (**d**) 1248 K; (**e**) 1263 K; (**f**) 1278 K; (**g**) 1293 K.

In summary, the modified Hensel–Spittel (mHS) equation is established, which is shown in Equation (47). The material parameters are listed in Table 4. The predicted stress the original Hensel–Spittel (oHS) equation and the modified Hensel–Spittel (mHS) equation is shown in Figure 14.

## 4. Discussion

In the present study, it was established two original and two modified constitutive equations. The two original constitutive equations are the original strain-compensated Arrhenius-type (os–cA-type) equation, and the original Hensel–Spittel (oHS) equation, respectively. Moreover, the two modified constitutive equations include the modified strain-compensated Arrhenius-type (ms–cA-type) equation and the modified Hensel–Spittel (mHS) equation.

A comparative study was made on the above four constitutive equations and their predictability was evaluated in terms of the correlation coefficient (R), average absolute relative error (AARE) and the relative error (RE) and the average root mean square error (RMSE). The correlation coefficient (R) is shown in Equation (48).

(48) $$R=\frac{\sum_{i=1}^{N}\left(E_{i}-\bar{E})(P_{i}-\bar{P}\right)}{\sqrt{\sum_{i=1}^{N}{\left(E_{i}-\bar{E}\right)}^{2}{\left(P_{i}-\bar{P}\right)}^{2}}}$$

where *E_i_* is the measured stress, *P_i_* is the estimated stress, $\bar{E}$ and $\bar{P}$ are the mean values of measured and estimated flow stresses, respectively.

**Figure 15:**
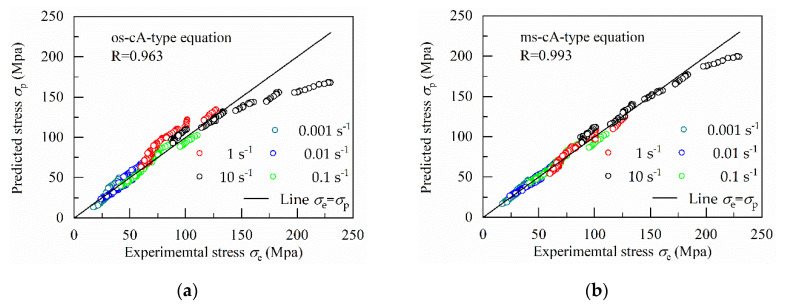

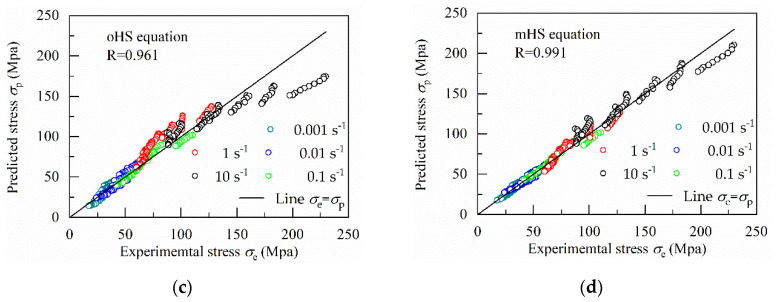
Correlation between the experimental stress and predicted flow stress values from (**a**) original strain-compensated Arrhenius-type (os–cA-type) equation; (**b**) modified strain-compensated Arrhenius-type (ms–cA-type) equation; (**c**) original Hensel–Spittel (oHS) equation; (**d**) modified Hensel–Spittel (mHS) equation.

The correlation coefficient (R) is a statistical measure of the strength of the linear relationship between two variables. It is assumed that the predicted stress *σ*_p_ is equal to the experimental stress *σ*_e_. There will be a linear relationship between *σ*_p_ and *σ*_e_, namely *σ*_p_ = *σ*_e_, when *σ*_p_ = *σ*_e_, R = 1. As the prediction accuracy decreases, the linear relationship between *σ*_p_ and *σ*_e_ becomes weaker and the value of R also decreases. Therefore, the correlation coefficient (R) can be used as a measure of prediction accuracy by verifying the strength of the linear relationship between *σ*_p_ and *σ*_e_. Figure 15 shows the correlation between the experimental stress and predicted flow stress values from the different constitutive equations.

In Figure 15, the R-value of the modified strain-compensated Arrhenius-type (ms–cA-type) equation (0.993) are the maximum, which is followed by the modified Hensel–Spittel (mHS) equation (0.991), the original strain-compensated Arrhenius-type (os–cA-type) equation (0.963) and the original Hensel–Spittel (oHS) equation (0.961).

Meanwhile, the straight line *σ*_p_ = *σ*_e_ are introduced into Figure 15, which is used to show the difference between these constitutive equations. When *σ*_p_ = *σ*_e_, the data point is located on the straight line *σ*_p_ = *σ*_e_. The distance from the data point to line *σ*_p_ = *σ*_e_ increases as the error value between *σ*_p_ and *σ*_e_ increases.

From Figure 15, it is easily found that the data point under the strain rate of 10 s^−1^ has the farthest distance to the straight line *σ*_p_ = *σ*_e_, which means the corresponding error is the largest. However, the flow stress is different as temperatures, strain and strain rates changes. The same error between the experimental and predicted stress may mean different results for two unequal experimental stress values. In statistics, normalization can overcome the above shortcomings. Therefore, as a normalized unbiased statistical parameter, the average absolute relative error (AARE) is used to verify the predictability of the four constitutive equations. The smaller the value of AARE, the higher the accuracy of the constitutive equation.

The modified strain-compensated Arrhenius-type equation has the smallest AARE-value (4.67%) when compared to the AARE-value of the modified Hensel–Spittel (mHS) equation (5.31%), the AARE-value of the original strain-compensated Arrhenius-type (os–cA-type) equation (10.16%), the AARE-value of the original Hensel–Spittel (oHS) equation (10.65%). The AARE-value of any original constitutive equation (namely, the os–cA-type equation and the oHS equation) is more than twice that of the corresponding modified constitutive equation (namely, the ms–cA-type equation and the mHS equation).

**Figure 16:**
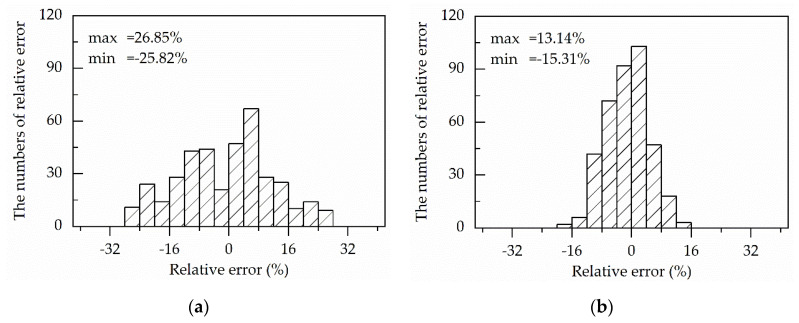

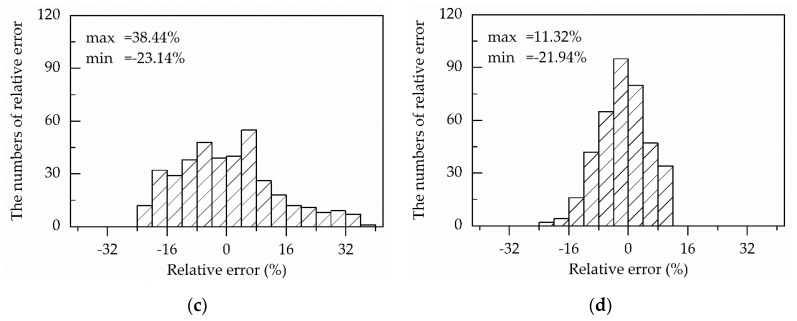
Statistical analysis of the relative error from (**a**) the original strain-compensated Arrhenius-type (os–cA-type) equation; (**b**) modified strain-compensated Arrhenius-type (ms–cA-type) equation; (**c**) original Hensel–Spittel (oHS) equation; (**d**) modified Hensel–Spittel (mHS) equation.

As a statistical parameter, the relative error is commonly employed to show the distribution of errors between the experimental and predicted values [6]. The relative error is calculated by comparing the data points and predictions via a term-by-term, which is shown in Equation (49).

(49) $$\mathrm{Relative}\;\mathrm{error}=\frac{\left(E_{i}-P_{i}\right)}{E_{i}}\times 100\%$$

where *E_i_* and *P_i_* still are the experimental data and is the predicted value, respectively. Moreover, the results are shown in Figure 16.

The relative errors gained from modified strain-compensated Arrhenius-type (ms–cA-type) equation and the modified Hensel–Spittel (mHS) equation vary from −15.31% to 13.14% and −21.94% to 11.32%, respectively, whereas it is in the range of −25.82% to 26.85% for the modified strain-compensated Arrhenius-type (ms–cA-type) equation and −23.14% to 38.44% for the modified Hensel–Spittel (mHS) equation.

Moreover, 90.65% and 87.80% of the numbers of relative errors of the modified strain-compensated Arrhenius-type (ms–cA-type) equation and the modified Hensel–Spittel (mHS) equation locate between RE-values of −10% and 10%. Compared with the two modified constitutive equation, the percentage of the RE-number of the original strain-compensated Arrhenius-type (os–cA-type) equation and the original Hensel–Spittel (oHS) equation are 57.40% and 55.84%, respectively, in the same RE-values range from −10% to 10%.

In addition, the average root mean square error (RMSE) is a statistical measure for model performance evaluation [14,38], which is used to compare the four constitutive equations further. The expression of RMSE is as follows:

(50) $$\mathrm{RMSE}=\sqrt{\frac{1}{N}\sum_{i=1}^{N}{\left(E_{i}-P_{i}\right)}^{2}}$$

where RMSE is the average root mean square error, *E_i_* is the experimental data and *P_i_* is the predicted value obtained from the model, and *N* is the total number of data employed in the investigation. Regarding the four constitutive equations, the results of RMSE-values are very similar to the results of ARRE-values. The RMSE-value of any original constitutive equation (namely, the os–cA-type equation and the oHS equation) is more than twice as big as the corresponding modified corresponding constitutive equation (namely, the ms–cA-type equation and the mHS equation).

In addition, RMSE-values and AARE-values under a single strain rate can be used to explain the effect of stain rates on the prediction accuracy of the four constitutive equations.

**Figure 17:**
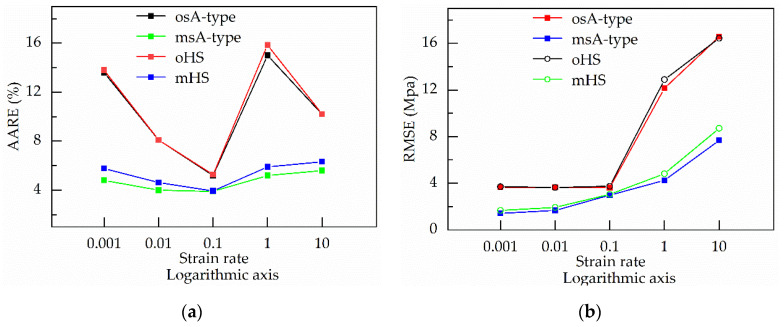
The relationship between the strain rate and (**a**) AARE; (**b**) RMSE obtained by the original strain-compensated Arrhenius-type (os–cA-type) equation, the modified strain-compensated Arrhenius-type (ms–cA-type) equation, the original Hensel–Spittel (oHS) equation and the modified Hensel–Spittel (mHS) equation.

From Figure 17, with increasing the strain rate, RMSE-values of the two original constitutive equation (namely, the os–cA-type equation and the oHS equation) change little in the beginning, but their AARE-values decrease sharply. The reason is that the stress increases with the increase of strain rate, which is shown in Figure 1. When the strain rate reaches 0.1 s^−1^, every original constitutive equation obtains the smallest AARE-value, which is slightly higher than the AARE-value of the any modified constitutive equations (namely, the ms–cA-type equation and the mHS equation). As the stain rate continues increasing, RMSE-values of the two original constitutive equations increase sharply, but their AARE-values increases first and then decreases. Because the increase ratio of RMSE is higher first and then lower than that of the experimental stress, and the values of stress rise sharply as the value of the strain rate increases from 1 to 10 s^−1^, which is shown in Figure 1. AARE-values of the two original constitutive equations reaches the maximum when the strain rate is one per second.

Although RMSE-values of the two modified constitutive equations (namely, the ms–cA-type equation and the mHS equation) also increase as the strain rate increases, their AARE-values change relatively little. The reason is that the RMSE-value of the two modified constitutive equation has the increasing ratio similar to the experimental stress. Moreover, under various strain rates, RMSE-value of every modified constitutive equation is lower than that of any original constitutive equation. Therefore, the modified constitutive equations can have higher accuracy and reflect the effect of strain rate on stress more accurately.

In sum, the results of the R, AARE, RMSE and RE of the four constitutive equations are listed in Table 5. Based on the above result, it is found that the original strain-compensated Arrhenius-type (os–cA-type) equation and the original Hensel–Spittel (oHS) equation have a similar and relatively lower prediction accuracy. The prediction accuracy of the modified strain-com Arrhenius-type constitutive equation is the highest, which is slightly higher than the prediction accuracy of the modified Hensel–Spittel (mHS) equation.

## 5. Conclusions

The hot deformation behavior of TC4–DT alloy was studied. For predicting the flow stress of the TC4–DT alloy, the modified strain-compensated Arrhenius-type equation and the modified Hensel–Spittel (mHS) equation were developed by revising strain rates and combining regression analysis and iterative methods. Meanwhile, the original strain-compensated Arrhenius-type equation and the original Hensel–Spittel (oHS) equation were established by the original linear regression methods. A comparative study was made on the above four equations, and the following conclusions were as follows:

(1) Both the original and modified strain-compensated Arrhenius-type (os–cA-type) equation and the original Hensel–Spittel (oHS) equation had a similar and relatively lower prediction accuracy, with R-value, AARE-value and RMSE-value of 0.963, 10.16% and 7.93 Mpa for the os–cA-type equation and of 0.961, 10.65% and 8.08 Mpa for the oHS equation;
(2) The modified strain-compensated Arrhenius-type (ms–cA-type) equation had the highest prediction accuracy, which had the highest R-value (0.993), the lowest AARE-value (4.67%) and MRSE-value (3.60 Mpa). The prediction accuracy of the modified Hensel–Spittel (mHS) equation was very close to that of the ms–cA-type equation. The R-value, AARE-value and RMSE-value of the mHS equation were 0.991, 5.31% and 4.04 Mpa, respectively. Regarding AARE and RMSE, the value of any modified constitutive equation was less than half the value of the corresponding original constitutive equation;
(3) Regarding the two modified constitutive equation (namely, the mHS equation and the ms–cA-type equation), AARE-value under different strain rated was lower, and its fluctuation was relatively small as the strain rate changed. The AARE-values of the original constitutive equation (namely, the oHS equation and the os–cA-type equation) in different strain rated were relatively higher and differ greatly. The result means the new modified constitutive equation was more precise to describe the relationship between the strain rate and stress.

## Figures and Tables

**Table 1:** Coefficients of the polynomial for *α*, *n*, Q and lnA.

*α*	*n*	Q	lnA
*a*_0_ = 0.0136	*N*_0_ = 5.024	Q_0_ = 425.153	*A*_0_ = 39.642
*a*_1_ = 0.0236	*N*_1_ = −5.757	Q_1_ = 9463.639	*A*_1_ = 860.817
*a*_2_ = −0.3988	*N*_2_ = 72.989	Q_2_ = −113,579.0	*A*_2_ = −10,345.921
*a*_3_ = 2.9127	*N*_3_ = −649.946	Q_3_ = 647,939.428	*A*_3_ = 58,956.037
*a*_4_ = −10.4516	*N*_4_ = 2802.387	Q_4_ = −2.029 × 10^−6^	*A*_4_ = −184,298.5
*a*_5_ = 19.8532	*N*_5_ = −6137.141	Q_5_= 3.575 × 10^−6^	*A*_5_ = 324,376.105
*a*_6_ = −19.0393	*N*_6_ = 6620.972	Q_6_= −3.332 × 10^−6^	*A*_6_ = −302,056.1
*a*_7_ = 7.2107	*N*_7_ = −2796.638	Q_7_ = 1.279 × 10^−6^	*A*_7_ = 115,906.977

**Table 2:** Coefficients of the polynomial for *α*, *n*, Q and lnA in the ms–cA-type equation.

ln*α*	*n*	Q	lnA	$\ln{\dot{\varepsilon }}^{*}$
*a*_0_ = −33.2764	*N*_0_ = 5.539	Q_0_ = 168.423	*A*_0_ = 181.345	*E*_0_ = −0.0769
*a*_1_ = 683.8600	*N*_1_ = 26.621	Q_1_ = 14,166.731	*A*_1_ = −2463.403	*E*_1_ = 0.9819
*a*_2_ = −7783.1519	*N*_2_ = −278.626	Q_2_ = −161,429.97	*A*_2_ = 28,124.806	
*a*_3_ = 45,443.6811	*N*_3_ = 1260.164	Q_3_ = 904,869.178	*A*_3_ = −169,711.35	
*a*_4_ = −147,592.0013	*N*_4_ = −3006.507	Q_4_= −2.811 × 10^−6^	*A*_4_ = 569,436.405	
*a*_5_ = 269,494.6845	*N*_5_ = 3912.161	Q_5_= 4.936 × 10^−6^	*A*_5_ = −1.068 × 10^−6^	
*a*_6_ = −258,493.9747	*N*_6_ = −2643.084	Q_6_= −4.593 × 10^−6^	*A*_6_ = 1.046 × 10^−6^	
*a*_7_ = 101,249.5269	*N*_7_ = 743.522	Q_7_ = 1.763 × 10^−6^	*A*_7_ = −415,487.31	

**Table 3:** Material parameters obtained by the traditional method.

A	*m*_1_	*m*_2_	*m*_3_	*m*_4_	*m*_5_	*m*_6_
3,199,230.981	−0.0113	0.1064	0.2329	0.0114	−0.0008	0.5115

**Table 4:** Material parameters in the modified Hensel–Spittel (mHS) equation.

*α*	A	*m*_1_	*m*_2_	*m*_3_	*m*_4_	*m*_5_	*m*_6_	*e*	*e*_1_
0.0003	342.914	−0.0075	−0.0863	0.16784	−0.0131	−0.0005	0.2511	2.7183	−0.0863

**Table 5:** R, AARE, RMSE and RE of the original strain-compensated Arrhenius-type (os–cA-type) equation, the modified strain-compensated Arrhenius-type (ms–cA-type) equation, the original Hensel–Spittel (oHS) equation and the modified Hensel–Spittel (mHS) equation.

Constitutive Equation	R	AARE (%)	RMSE (Mpa)	Max of RE (%)	Min of RE (%)
oHS	0.961	10.65	8.08	38.44	−23.14
os–cA-type	0.963	10.16	7.93	26.85	−25.82
mHS	0.991	5.31	4.04	11.32	−21.94
ms–cA-type	0.993	4.67	3.60	13.14	−15.31

## Appendix A

**Table A1:** Experimental stress under different deforming conditions (Mpa).

T(K)	Strain Rate (s^−1^)	Strain										
		0.1	0.15	0.2	0.25	0.3	0.35	0.4	0.45	0.5	0.55	0.6
1203	0.001	40.19	39.59	38.40	36.61	36.01	35.42	33.63	32.44	31.84	30.65	30.65
1203	0.01	65.21	64.02	62.23	60.45	59.26	59.26	57.47	55.68	54.49	52.70	51.51
1203	0.1	110.50	108.12	105.74	105.14	103.35	100.97	99.78	98.58	97.39	96.20	95.01
1203	1	127.19	127.19	126.00	124.21	123.02	121.23	120.63	119.44	118.25	116.46	116.46
1203	10	229.68	227.90	227.90	224.32	219.55	214.79	210.61	207.04	202.27	200.48	197.50
1218	0.001	38.16	36.48	34.81	33.13	33.13	32.01	30.34	29.78	29.22	28.66	28.66
1218	0.01	60.52	58.84	55.49	54.37	52.13	49.90	48.22	47.10	45.99	45.43	44.31
1218	0.1	85.11	83.99	81.76	78.96	77.29	75.61	74.49	74.49	74.49	73.93	75.05
1218	1	101.32	101.32	101.32	100.76	100.76	100.76	100.76	99.09	100.76	99.64	100.76
1218	10	182.93	183.49	181.25	181.81	181.25	179.01	176.22	175.10	173.42	172.31	172.31
1233	0.001	35.94	35.42	33.85	31.77	31.25	30.73	30.21	28.65	27.08	26.04	26.04
1233	0.01	54.69	53.13	51.56	50.00	48.96	46.88	45.31	44.79	42.71	41.67	40.10
1233	0.1	76.04	75.52	73.96	72.40	71.88	71.35	70.83	69.79	69.79	69.79	69.79
1233	1	91.67	93.23	91.67	90.63	89.58	88.02	85.94	84.38	82.81	83.33	82.81
1233	10	158.85	160.94	158.85	156.25	157.29	151.56	151.56	148.44	147.40	145.31	144.79
1248	0.001	32.73	31.64	30.55	29.82	28.36	28.00	26.55	25.82	24.73	24.00	23.64
1248	0.01	52.36	51.27	49.45	48.00	46.55	44.36	43.27	41.82	40.00	38.91	38.18
1248	0.1	68.00	67.27	65.82	65.82	65.45	64.36	63.27	63.27	62.91	62.55	60.36
1248	1	79.64	79.27	78.55	78.18	77.45	76.36	74.18	73.82	72.36	72.36	71.27
1248	10	133.45	133.09	134.55	133.45	133.09	131.64	129.82	127.27	125.82	125.82	125.45
1263	0.001	29.53	28.41	27.65	26.15	25.78	25.03	24.28	23.90	23.15	22.40	22.03
1263	0.01	46.42	44.92	43.04	41.16	40.04	38.16	37.04	36.66	35.53	35.16	35.16
1263	0.1	62.93	62.18	61.80	61.05	60.30	59.93	58.05	57.30	56.55	55.42	54.30
1263	1	73.06	73.81	73.81	72.68	71.93	70.43	68.18	67.43	66.68	66.68	65.93
1263	10	123.34	126.72	125.22	123.34	121.09	120.71	118.84	118.09	116.59	115.46	113.96
1278	0.001	26.59	25.25	24.92	23.58	23.24	22.57	22.23	21.90	20.89	21.56	21.23
1278	0.01	39.66	37.99	36.65	34.64	33.63	32.63	31.62	30.95	30.61	29.61	29.27
1278	0.1	56.76	56.09	55.42	56.09	54.41	53.41	51.06	51.06	49.05	48.04	47.71
1278	1	68.83	69.50	69.50	68.49	67.82	66.82	66.48	64.80	64.80	63.13	63.13
1278	10	98.32	100.34	101.01	99.66	98.66	97.65	96.65	95.64	94.30	92.96	93.63
1293	0.001	22.89	23.42	21.29	21.83	19.96	19.43	19.96	18.90	18.37	17.30	17.03
1293	0.01	34.07	33.80	31.94	30.87	28.75	28.21	26.88	26.35	25.02	24.22	23.95
1293	0.1	50.30	50.04	49.51	47.38	46.58	47.64	44.45	43.12	43.12	42.32	41.52
1293	1	68.14	67.34	66.27	65.74	65.48	63.61	63.08	62.02	60.15	60.68	59.89
1293	10	87.30	89.43	89.96	92.89	90.23	89.16	88.90	89.70	88.37	88.63	88.63
